# Dasatinib suppresses particulate-induced pyroptosis and acute lung inflammation

**DOI:** 10.3389/fphar.2023.1250383

**Published:** 2023-08-29

**Authors:** Yixi Pan, Kenta Ikoma, Risa Matsui, Akiyoshi Nakayama, Naoki Takemura, Tatsuya Saitoh

**Affiliations:** ^1^ Laboratory of Bioresponse Regulation, Graduate School of Pharmaceutical Sciences, Osaka University, Osaka, Japan; ^2^ Department of Integrative Physiology and Bio-Nano Medicine, National Defense Medical College, Saitama, Japan; ^3^ Global Center for Medical Engineering and Informatics, Osaka University, Osaka, Japan; ^4^ Center for Infectious Diseases for Education and Research (CiDER), Osaka University, Osaka, Japan

**Keywords:** Src family kinases, dasatinib, particulates, pyroptosis, interleukin-1 alpha

## Abstract

**Background:** Humans are constantly exposed to various industrial, environmental, and endogenous particulates that result in inflammatory diseases. After being engulfed by immune cells, viz. Macrophages, such particulates lead to phagolysosomal dysfunction, eventually inducing pyroptosis, a form of cell death accompanied by the release of inflammatory mediators, including members of the interleukin (IL)-1 family. Phagolysosomal dysfunction results in the activation of the nod-like receptor family pyrin domain containing 3 (NLRP3) inflammasome, an immune complex that induces pyroptosis upon exposure to various external stimuli. However, several particulates induce pyroptosis even if the NLRP3 inflammasome is inhibited; this indicates that such inhibition is not always effective in treating diseases induced by particulates. Therefore, discovery of drugs suppressing particulate-induced NLRP3-independent pyroptosis is warranted.

**Methods:** We screened compounds that inhibit silica particle (SP)-induced cell death and release of IL-1α using RAW264.7 cells, which are incapable of NLRP3 inflammasome formation. The candidates were tested for their ability to suppress particulate-induced pyroptosis and phagolysosomal dysfunction using mouse primary macrophages and alleviate SP-induced NLRP3-independent lung inflammation.

**Results:** Several Src family kinase inhibitors, including dasatinib, effectively suppressed SP-induced cell death and IL-1α release. Furthermore, dasatinib suppressed pyroptosis induced by other particulates but did not suppress that induced by non-particulates, such as adenosine triphosphate. Dasatinib reduced SP-induced phagolysosomal dysfunction without affecting phagocytosis of SPs. Moreover, dasatinib treatment strongly suppressed the increase in IL-1α levels and neutrophil counts in the lungs after intratracheal SP administration.

**Conclusion:** Dasatinib suppresses particulate-induced pyroptosis and can be used to treat relevant inflammatory diseases.

## Introduction

The prevalence of inflammatory diseases caused by environmental pollution, overnutrition, and aging is increasing in, both, developed and developing countries (Barquera et al., 2015; Lopez and Kuller, 2019; von Schneidemesser et al., 2020; Kimura et al., 2021). Therefore, there is an urgent need to address these global health problems. Particulate irritants that result in aberrant inflammation by stimulating immune cells are involved in the pathogenesis of modern diseases. For example, the inhalation of particulates comprising crystalline silica, such as yellow dust and PM_2.5_, results in pneumoconiosis and allergies (Dostert et al., 2008; Kuroda et al., 2016). Amorphous silica can also lead to pneumoconiosis (Cho et al., 2007). Moreover, the ingestion of microplastics, a recent cause of serious marine pollution, induces liver injury (Mu et al., 2022). Industrial materials such as asbestos and carbon nanotubes lead to pneumoconiosis and mesothelioma, whereas titanium dioxide causes dermatitis and enteritis (Dostert et al., 2008; Yazdi et al., 2010; Palomaki et al., 2011). In addition, metabolic crystalline particles, including monosodium urate (MSU), cholesterol, and calcium oxalate crystals, generated in the body owing to overnutrition, cause gout, arteriosclerosis, and nephritis, respectively (Martinon et al., 2006; Duewell et al., 2010; Mulay et al., 2012). Harmful particulates also include protein aggregates, such as amyloid-β deposits, which lead to Alzheimer’s disease with aging (Halle et al., 2008). Therefore, there is an urgent need to develop effective therapeutic strategies by elucidating particulate-induced inflammatory responses.

The immune system is the host’s defense mechanism against infection. It induces the production of inflammatory mediators by sensing pathogens using pattern recognition receptors thereby triggering inflammation for their clearance (Takeuchi and Akira, 2010). In contrast, the immune system also induces inflammation in response to particulate irritants, leading to tissue injury and dysfunction. Particulates induce immune cell pyroptosis, a form of cell death accompanied by the release of inflammatory mediators, particularly members of the interleukin (IL)-1 family (Hornung et al., 2008; Yang et al., 2019). Drugs that prevent pyroptosis can potentially treat inflammatory diseases induced by particulate irritants.

Invading or intrinsic particulates are engulfed by immune cells such as macrophages. Phagosomes containing foreign substances undergo maturation by sequentially recruiting various proteins, including Rab guanosine triphosphatase, and eventually fusing with lysosomes to form phagolysosomes, which digest the engulfed substances (Kinchen and Ravichandran, 2008). However, particulates with sharp crystal structures and/or particular surface properties destabilize the phagolysosomal membrane, resulting in the leakage of its contents (Hornung et al., 2008). Phagolysosomal dysfunction activates the nod-like receptor family pyrin domain containing 3 (NLRP3), an intracellular pattern recognition receptor, which senses microbial invasion (Yang et al., 2019). Upon activation, NLRP3 forms a protein complex (termed inflammasome) with the adaptor molecule apoptosis-associated speck-like protein containing a caspase recruitment domain and caspase-1 (Yang et al., 2019). Caspase-1 induces the maturation of cytokines of the IL-1 family, such as IL-1β and IL-18, and gasdermin D, which form pores in the plasma membrane and induce cytokine release and subsequent cell death (Yang et al., 2019). To date, drugs targeting the NLRP3 inflammasome and its downstream cytokines have been developed to treat pyroptosis-induced inflammatory diseases (Yang et al., 2019). However, recent studies suggest that targeting NLRP3 inflammasome-associated responses are insufficient in treating inflammatory diseases induced by particulates, such as silica particles (SPs) and MSU, because these particulates continue to induce cell death and inflammatory mediator release in the absence of NLRP3 or gasdermin D (Yazdi et al., 2010; Groß et al., 2012; Rabolli et al., 2014; Kuroda et al., 2016; Rashidi et al., 2019; Ikoma et al., 2022). IL-1α is an important molecule among those released during NLRP3-independent cell death owing to its proinflammatory effects by binding to the same receptor as IL-1β. Several animal studies have reported that IL-1α plays critical roles in the development of particulate-induced inflammatory diseases (Yazdi et al., 2010; Groß et al., 2012; Rabolli et al., 2014; Kuroda et al., 2016; Ikoma et al., 2022). However, preventing NLRP3-independent cell death and the resultant release of inflammatory mediators remains a key challenge during the development of efficient treatment strategies for inflammatory diseases induced by particulate irritants.

In a previous study, we proposed the natural compound oridonin as an effective drug candidate for treating particulate-induced inflammatory diseases (Ikoma et al., 2022). Oridonin suppressed particulate-induced NLRP3-independent cell death and IL-1α release and attenuated NLRP3-independent lung inflammation in a mouse model of silicosis. However, oridonin and its derivatives are still not clinically approved. In the present study, we performed a library screening via drug repositioning to identify compounds that can suppress particulate-induced cell death and IL-1α release. Consequently, we identified dasatinib, a clinically approved drug, as a potential therapeutic agent for particulate-induced inflammatory diseases.

## Materials and methods

### Reagents and cell lines

Pfizer and Food and Drug Administration (FDA)-approved drug libraries were kindly provided by the Center for Supporting Drug Discovery and Life Science Research, Graduate School of Pharmaceutical Sciences, Osaka University. Lipopolysaccharide (LPS) from *Escherichia coli* O111:B4 was purchased from Invivogen (San Diego, CA, United States). Plain and fluorescent amorphous SPs (Sicastar; 500, 1,500, and 3,000 nm in diameter) were purchased from MicroMod (Rostock, Germany). Chemi-Lumi One Super and MSU were purchased from Nacalai Tesque (Kyoto, Japan). Gobi Kosa dust (yellow dust) was purchased from the National Institute for Environmental Studies (Ibaraki, Japan). Adenosine triphosphate (ATP) was purchased from Enzo Life Sciences (Farmingdale, NY, United States). DRAQ7 was purchased from Biostatus (Loughborough, United Kingdom). Bosutinib, cytochalasin D (Cyto D), dasatinib, L-leucyl-L-leucine methyl ester (LLoMe), PD-161570, and iFluor-488-conjugated phalloidin were purchased from Cayman Chemical Company (Ann Arbor, Michigan, United States). PD-166285 dihydrochloride was purchased from Tocris Biosciences (Abingdon, United Kingdom). Dasatinib hydrochloride for *in vivo* experiments was purchased from MedChem Express (Monmouth Junction, NJ, United States). Hoechst 33342, LysoTracker Deep Red, and ProLong Gold antifade Mountant were purchased from Thermo Fisher Scientific (Waltham, MA, United States). DRAQ5 and the enzyme-linked immunosorbent assay (ELISA) kits for mouse IL-1β were purchased from BioLegend (San Diego, CA, United States). Can Get Signal Immunoreaction Enhancer Solution was purchased from Toyobo (Osaka, Japan). Immobilon Forte Western horseradish peroxidase (HRP) substrate was purchased from Merck Millipore (Burlington, MA, United States). Collagenase (crude type) was purchased from FUJIFILM Wako Pure Chemical Corporation (Osaka, Japan) and DNase I was purchased from Sigma-Aldrich (St. Louis, MO, United States). The ELISA kits for mouse chemokine (C-X-C motif) ligand (CXCL) 1 and mouse and human IL-1α and IL-1β were purchased from R&D Systems (Minneapolis, MN, United States). The cytotoxicity lactate dehydrogenase (LDH) assay kit (WST) was purchased from Dojindo Laboratories (Kumamoto, Japan).

RAW264.7, a mouse macrophage cell line, and THP-1, a human monocytic cell line, were purchased from Riken (Ibaraki, Japan).

### Antibodies

Anti-phospho-Src family (D49G4), anti-RAB5A, member RAS oncogene family (RAB5A) (E6N8S), HRP-conjugated anti-mouse IgG, and HRP-conjugated anti-rabbit IgG antibodies were purchased from Cell Signaling Technology (Danvers, MA, United States). Anti-lysosome-associated membrane protein-1 (LAMP-1) (1D4B) and anti-phosphatidylinositol 4,5-bisphosphate (PIP2) (2C11) antibodies were purchased from Abcam (Cambridge, United Kingdom). Anti-actin antibody (C-11) was purchased from Santa Cruz Biotechnology (Dallas, TX, United States). Alexa Fluor-labeled secondary antibodies and HRP-conjugated anti-goat IgG (H + L) antibodies were purchased from Thermo Fisher Scientific. The Alexa Fluor 488-conjugated anti-mouse CD11b (M1/70), Pacific Blue-conjugated anti-mouse CD45 (30-F11), allophycocyanin-conjugated anti-mouse CD68 (FA-11), fluorescein isothiocyanate-conjugated anti-mouse CD80 (16-10A1), phycoerythrin-cyanine7-conjugated anti-mouse CD86 (GL-1), Alexa Fluor 647-conjugated anti-mouse Ly-6G (1A8), phycoerythrin-cyanine7-conjugated anti-mouse Ly-6C (HK1.4), and phycoerythrin-conjugated anti-mouse major histocompatibility complex class II (M5/114.15.2) antibodies were purchased from BioLegend.

### MSU crystal formation

MSU crystals were prepared using a previously described method (Ikoma et al., 2022).

### Mice

C57BL/6J mice (5-week-old females) were purchased from Japan SLC, Inc. (Shizuoka, Japan). During the experimental period, all mice were housed in standard cages in a temperature-controlled room under a 12-h light/dark cycle at the animal care facility of the Graduate School of Pharmaceutical Sciences, Osaka University. Mice were provided *ad libitum* access to standard laboratory mouse chow and drinking water.

### Screening of compounds that can inhibit particulate-induced cell death and IL-1α release

RAW264.7 cells were seeded at a density of 3.5 × 10^4^ cells/well into glass-bottom 96-well plates and primed with LPS (100 ng/mL) for 16 h in Eagle’s minimal essential medium supplemented with 10% fetal calf serum (FCS), penicillin (100 U/mL), streptomycin (100 μg/mL), and non-essential amino acids (Nacalai Tesque, 100x). Cells were pretreated with each compound (5 µM) from the Pfizer- and FDA-approved drug libraries for 30 min and then stimulated with SPs (500 nm in diameter, 500 μg/mL) in the presence of DRAQ7 (2 µM) and Hoechst 33342 (1 μg/mL) for 2 h. After the supernatants were collected, the cells were fixed with 4% paraformaldehyde for 15 min and then rinsed with ice-cold phosphate buffered saline. Images were acquired from four fields per well using the Cell Voyager CV8000 High Content Screening System (Yokogawa Electric Corp., Tokyo, Japan). Hoechst 33342-positive and DRAQ7-negative cells and Hoechst 33342 and DRAQ7 double-positive cells were considered viable and dead cells, respectively. The cell death rate was calculated by dividing the number of dead cells by the total number of cells using CellPathfinder software (Yokogawa Electric Corp.). IL-1α levels in the cell culture supernatants were measured using ELISA, as described below. A test compound was defined as a hit if the mean percentage inhibition of cell death and IL-1α release was >50%.

### Macrophage preparation and stimulation

To prepare bone marrow-derived macrophages (BMDMs), mouse bone marrow cells were cultured with macrophage colony-stimulating factor (10 ng/mL) in Roswell Park Memorial Institute (RPMI) 1,640 supplemented with 10% FCS, penicillin, and streptomycin. 5 days after culture initiation, cells were collected and used as BMDMs. The BMDMs were seeded at a density of 4 × 10^5^ cells/well in 48-well plates and primed with LPS (200 ng/mL) in RPMI1640 supplemented with 10% FCS for 6 h. The primed cells were pretreated with bosutinib (20 µM), Cyto D (20 µM), dasatinib (20 µM), PD-161570 (20 µM), or PD-166285 (20 µM) for 30 min and then stimulated with SPs (500, 1,500 or 3,000 nm in diameter, 300 μg/mL), MSU (300 μg/mL), yellow dust (500 μg/mL), or ATP (3 mM) for 2 h or with LLoMe (0.5 mM) for 3 h.

THP-1 cells were seeded at a density of 4 × 10^5^ cells/well in 48-well plates and induced for macrophage differentiation by culturing them with phorbol 12-myristate 13-acetate (10 ng/mL) in RPMI1640 supplemented with 10% FCS for 1 day, followed by an additional 2 days of culture in phorbol 12-myristate 13-acetate-free medium. THP-1 cells were primed with LPS (50 ng/mL) in RPMI1640 supplemented with 10% FCS for 16 h. The primed cells were pretreated with dasatinib (20 µM) for 30 min and then stimulated with SPs (1,500 nm in diameter, 500 μg/mL) for 4 h.

The supernatants from each culture condition were collected after centrifuging the samples at 440 ×*g* and 4°C for 5 min. The supernatants and cell samples were analyzed.

### Cell viability measurement

Cell viability was determined by measuring LDH activity in the culture supernatants using the cytotoxicity LDH assay kit (WST) according to the manufacturer’s instructions.

### Immunoblotting

Cell samples were washed and lysed with 30 µL of the lysis buffer containing 62.5 mM Tris–HCl (pH 6.8 at 25°C), 2% (w/v) sodium dodecyl sulfate, 10% (v/v) glycerol, 0.01% (w/v) bromophenol blue, and 42 mM dithiothreitol. Samples were then processed for immunoblotting using a previously described method (Matsui et al., 2022). Each protein was probed with the appropriate antibodies listed in the subsection “Antibodies,” and the blots were visualized using Immobilon Forte Western HRP Substrate (Merck Millipore) or Chemi-Lumi One Super (Nacalai Tesque). Immunoreactive bands were detected using FUSION Solo S (Vilber Lourmat, Collégien, France) and quantified using the ImageJ software bundled with 64-bit Java 8. (Version 1.53t) (National Institutes of Health, MD, United States).

### Observation of particulate uptake by BMDMs

BMDMs were cultured on coverslips and primed with LPS (200 ng/mL) for 6 h. Further, they were pretreated with Cyto D (20 µM) or dasatinib (20 µM) for 30 min and stimulated with fluorescent SPs (1,500 nm in diameter, 20 μg/mL) for 2 h. The stimulated cells were fixed with 4% paraformaldehyde for 15 min and permeabilized with digitonin (50 μg/mL) for 15 min. The membrane-permeabilized cells were stained with iFluor-488-conjugate phalloidin (1000-fold dilution) and DRAQ5 (200 µM) for 30 min. Images were obtained using the Cell Voyager CV8000 High Content Screening system, and the number of phagocytosed SPs per cell was counted using the CellPathfinder software.

### Immunocytochemistry

BMDMs were cultured on coverslips and fixed with 3% paraformaldehyde. Immunocytochemistry was performed as described previously (Ikoma et al., 2022). Samples were visualized under an inverted fluorescence microscope (DMI6000B; Leica Microsystems, Wetzlar, Germany) and photographed using the Application Suite X software imaging system (Leica Microsystems).

### Induction of lung inflammation

Mice were randomly divided into four groups and anesthetized via isoflurane inhalation. The groups received different treatments as follows: 1) intratracheal administration of phosphate buffered saline; 2) intratracheal SP administration (100 mg/kg); 3) intratracheal SP administration (100 mg/kg) in combination with intragastric dasatinib administration (30 mg/kg); and 4) intratracheal administration of SP (100 mg/kg) in combination with dasatinib (10 mg/kg). Bronchoalveolar lavage (BAL) fluid was collected 12 h after the intratracheal administration of SPs. Subsequently, 1 mL of phosphate buffered saline was flushed into the lungs, with a recovery of approximately 700 µL of BAL fluid. The remaining lung tissues were used for isolating lung leukocytes as described below. In separate experiments, the lungs were collected 12 h after the intratracheal administration of SPs for histological analysis.

### Isolation of lung leukocytes

Lung tissues were minced using a pair of scissors and subsequently digested with collagenase (2 mg/mL) and DNase I (100 μg/mL) in RPMI1640 medium supplemented with 10% FCS with continuous stirring at 37°C for 60 min. The suspended cells were centrifuged at a density-gradient of 40%–80% (v/v) Percoll. Cells (lung leukocytes) were collected from the interface, washed, and then used in further experiments.

### Flow cytometric analysis

BMDMs were stained with LysoTracker Deep Red according to the manufacturer’s instructions. Lung leukocytes were stained with antibodies against mouse CD11b, CD45, CD68, CD80, CD86, Ly-6C, Ly-6G, and major histocompatibility complex class II, according to the manufacturer’s instructions. CD45^+^ CD11b^+^ Ly-6C^med^ Ly-6G^high^ cells were identified as neutrophils (Ikoma et al., 2022). CD45^+^ CD80^+^ CD86^+^ major histocompatibility complex class II^high^ CD68^+^ cells were identified as M1 macrophages (Supplementary Figure S1) (Haloul et al., 2019; Nascimento Da Conceicao et al., 2021). The data were acquired using a flow cytometer (CytoFLEX; Beckman Coulter, Brea, CA, United States) and analyzed using the FlowJo software (TreeStar, Ashland, OR, United States).

### Histological analysis

The mouse lungs were fixed for 24 h with 10% formalin and embedded in paraffin. Five-micrometer sections of the mouse lungs were stained with hematoxylin and eosin and observed under the BZ-X800 automated high-resolution microscope (Keyence, Osaka, Japan) with an analysis application.

### ELISA

Mouse CXCL1, mouse and human IL-1α and IL-1β levels in the culture supernatant and BAL fluid were measured using ELISA kits, according to the manufacturer’s instructions.

### Statistical analyses

All data was calculated using GraphPad Prism 8.0 software (Boston, MA, United States). One-way analysis of variance and the Tukey–Kramer *post hoc* test were performed for multiple group comparisons. Statistical significance was set at a *p*-value of <0.05.

## Results

### Dasatinib suppresses particulate-induced pyroptosis in macrophages

RAW264.7 cells are a macrophage cell line with impaired apoptosis-associated speck-like protein containing a caspase recruitment domain expression and negligible effects on the NLRP3 inflammasome (Pelegrin et al., 2008). These cells were used to identify inhibitors against particulate-induced NLRP3 inflammasome-independent pyroptosis. Candidate inhibitors were screened by elucidating whether the treatment of LPS-primed RAW264.7 cells with the test compounds before SP stimulation suppressed cell death and IL-1α release. SP-induced cell death was determined using CellVoyager CV8000, which allows high-throughput imaging-based screening. Two nuclear staining dyes, Hoechst 33342 and DRAQ7, were used to determine cell viability. Hoechst 33342 was used to stain the cell nuclei, whereas DRAQ7, a membrane-impermeable dye, was used to stain the nuclei of dead cells. Accordingly, Hoechst 33342-positive and DRAQ7-negative cells and Hoechst 33342 and DRAQ7 double-positive cells were considered viable and dead cells, respectively (Supplementary Figure S2A). Simultaneously, IL-1α levels in the culture supernatants were also measured. Screening of 1,240 compounds present in the Pfizer- and FDA-approved libraries identified two anticancer drugs, bosutinib and dasatinib, which suppressed SP-induced cell death and IL-1α release (Table 1; Supplementary Figures S2B, C). Similar results were obtained for PD-161570, PD-166285, PD-173952, and PD-407824. Interestingly, all the drug candidates targeted Src family kinases (SFKs), well-known signaling factors involved in various cellular functions, including the induction of inflammatory responses, cell proliferation, cell differentiation, and metabolism (Parsons and Parsons, 2004; Byeon et al., 2012).

**TABLE 1:** List of candidate drugs that inhibit particulate-induced cell death accompanied by interleukin (IL)-1α release.

Stimulation	Candidate drugs	Cell death (%)	IL-1α (pg/mL)	Reference
None	—	4 ± 0.2	201.5 ± 0.7	—
Silica particles	—	54.1 ± 3	3192.7 ± 36.2	—
	Bosutinib	3.3 ± 0.9	125.7 ± 35.4	Golas et al. (2003)
	Dasatinib	26.6 ± 4	1262.7 ± 766.7	Lombardo et al. (2004)
	PD-161570	7.9 ± 3.5	229.8 ± 0.1	Hamby et al. (1997)
	PD-166285	4.5 ± 1.4	143 ± 4.2	Panek et al. (1997)
	PD-173952	2.7 ± 1.3	718.4 ± 44.1	Dorsey et al. (2002)
	PD-407824	4.4 ± 0.6	1524.5 ± 261.7	Palmer et al. (2006)

**FIGURE 1:**
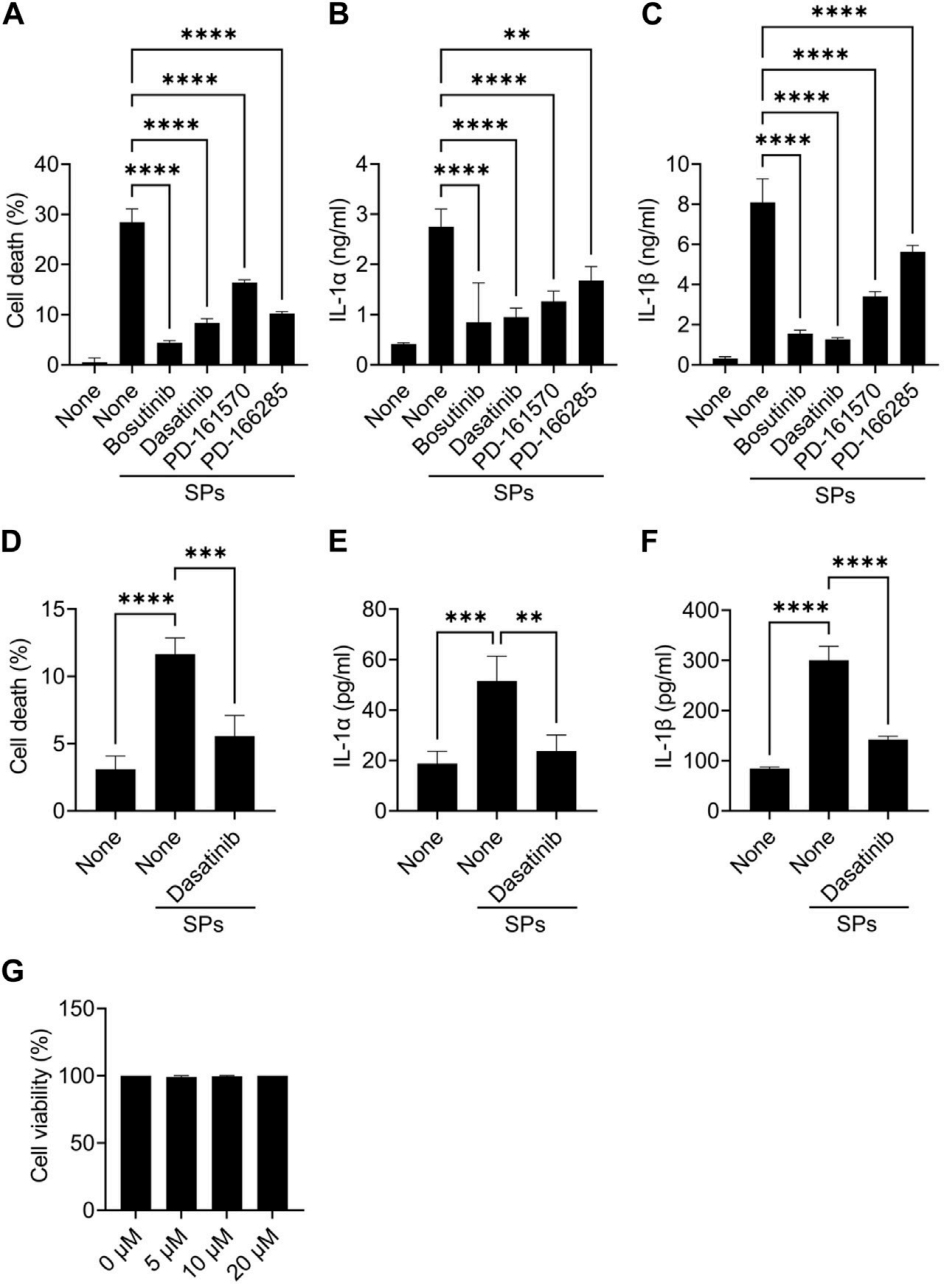
Dasatinib suppresses silica particle (SP)-induced cell death accompanied by interleukin-1 (IL-1) α release. **(A–C)** Bone marrow-derived macrophages (BMDMs) were primed with lipopolysaccharide (LPS) (200 ng/mL) for 6 h. The cells were then treated with 20 µM of bosutinib, dasatinib, PD-161570, or PD-166285 and were stimulated or not stimulated with SPs (1,500 nm in diameter, 300 μg/mL) for 2 h. **(A)** The cell death rate was determined by measuring lactate dehydrogenase (LDH) activity in the culture supernatant. **(B,C)** IL-1α and IL-1 beta (β) levels in the culture supernatants were measured using enzyme-linked immunosorbent assay (ELISA). **(D–F)** Phorbol 12-myristate 13-acetate-differentiated THP-1 cells were primed with LPS (50 ng/mL) for 16 h. The cells were then treated with dasatinib (20 µM) and were stimulated or not stimulated with SPs (1,500 nm in diameter, 500 μg/mL) for 4 h. **(D)** Cell death rate was determined by measuring LDH activity in the culture supernatant. **(D,E)** IL-1α and IL-1β levels in the culture supernatants were measured using ELISA. **(G)** BMDMs were treated with increasing doses of dasatinib (0–20 µM) for 12 h; thereafter, the cell death rate was determined by measuring LDH activity in the culture supernatant. The results are presented as the mean ± standard deviations (SD) of values from triplicate wells. **, *p* < 0.01; ***, *p* < 0.001; and ****, *p* < 0.0001.

Further, mouse primary macrophages were used to validate the effects of bosutinib, dasatinib, PD-161570, and PD-166285 (PD-173952 and PD-407824 were excluded because they are not approved yet and exhibit relatively weak suppressive effects against SP-induced pyroptosis). To assess whether these drugs inhibit SP-induced macrophage pyroptosis, we measured IL-1β and LDH release as indicators of pyroptosis, in addition to IL-1α release. LDH is released when the plasma membrane ruptures and is often used to measure the incidence of lytic cell death, including pyroptosis (Kayagaki et al., 2021). All the drug candidates significantly suppressed SP-induced cell death accompanied by IL-1α and IL-1β release in BMDMs (Figures 1A–C). Since dasatinib is clinically well-studied (Araujo and Logothetis, 2010), it was used as a representative of these candidates in subsequent experiments. We investigated whether dasatinib effectively suppresses SP-induced pyroptosis in human cells. Dasatinib was found to successfully suppress SP-induced cell death accompanied by IL-1α and IL-1β release in phorbol 12-myristate 13-acetate-treated macrophage-like THP-1 cells (Figures 1D–F). Furthermore, dasatinib did not affect the viability of BMDMs during the 12 h treatment at concentrations required to inhibit SP-induced pyroptosis (Figure 1G). Therefore, dasatinib and other SFK inhibitors effectively suppressed particulate-induced pyroptosis.

### Increase in active SFK levels enhance particulate-induced cell death

**FIGURE 2:**
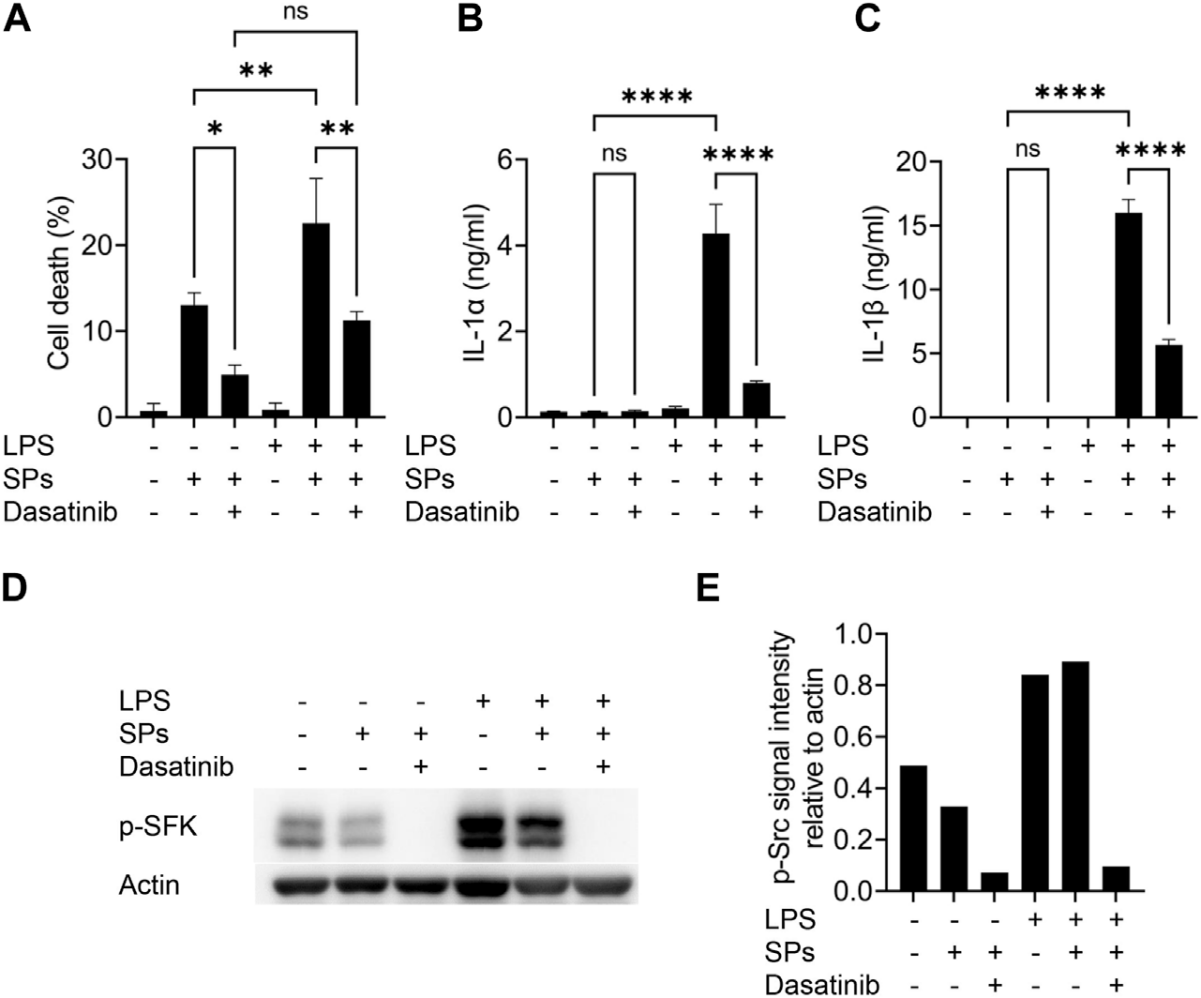
Lipopolysaccharide (LPS)-priming increases the levels of phosphorylated Src family kinases (p-SFKs) and enhances silica particle (SP)-induced pyroptosis. **(A–E)** Unprimed and LPS-primed bone marrow-derived macrophages (BMDMs) were treated with dasatinib (20 µM) and stimulated or not stimulated with SPs (1,500 nm in diameter, 300 μg/mL) for 2 h. **(A)** The cell death rate was determined by measuring lactate dehydrogenase (LDH) activity in the culture supernatants. **(B,C)** Interleukin-1 alpha (IL-1α) and IL-1 beta (β) levels in the culture supernatants were measured using enzyme-linked immunosorbent assay (ELISA). **(D)** Immunoblotting of p-SFKs in the cell extracts of BMDMs. **(E)** Quantification of p-SFK levels compared to actin control under conditions indicated in **(D)**. The results are presented as the mean ± SD of values from triplicate wells. *, *p* < 0.05; **, *p* < 0.01; and ****, *p* < 0.0001; ns, not significant.

Macrophages were initially primed with LPS to induce intracellular IL-1α and IL-1β expression by activating toll-like receptor 4. However, toll-like receptor 4 increases the levels of active SFKs by enhancing their transcription and phosphorylation at Tyr416 (Boggon and Eck, 2004; Maa and Leu, 2016). Thus, we determined whether SFKs mediate particulate-induced cell death without priming. As a result, dasatinib significantly suppressed SP-induced cell death in BMDMs even without LPS priming, although the unprimed cells did not release IL-1α and IL-1β upon SP stimulation (Figures 2A–C). Immunoblotting revealed the phosphorylation of SFKs at Tyr416 (phosphorylated SFKs; p-SFKs) in unprimed BMDMs (Figures 2D, E). Furthermore, LPS priming significantly enhanced SP-induced cell death of BMDMs and increased p-SFK levels. Interestingly, dasatinib decreased SP-induced cell death rates and p-SFK levels in LPS-primed BMDMs to levels comparable to those observed in dasatinib-treated unprimed BMDMs. SP stimulation tended to slightly decrease p-SFKs levels in unprimed BMDMs, but did not affect those in LPS-primed cells. To summarize, these results suggest that basal p-SFK levels can mediate particulate-induced cell death, and an increase in p-SFK levels via priming is associated with particulate-induced cell death.

### Dasatinib suppresses particulate-induced pyroptosis of various sizes or materials

**FIGURE 3:**
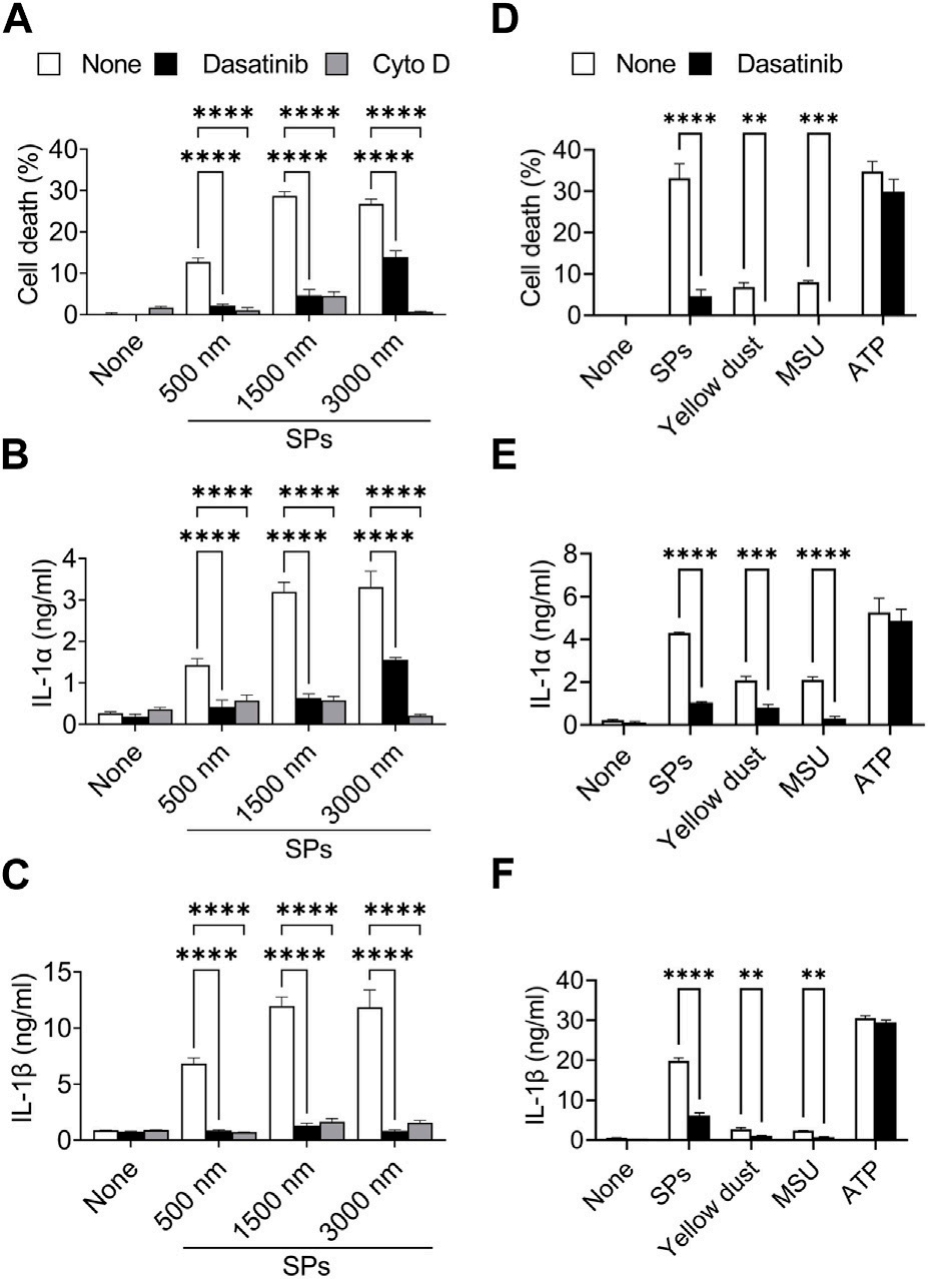
Dasatinib suppresses pyroptosis induced by particulates of various sizes or materials. **(A–C)** Primed bone marrow-derived macrophages (BMDMs) were treated with 20 µM dasatinib or cytochalasin D (Cyto D) and stimulated or not stimulated with silica particles (SPs) (500, 1,500, or 3,000 nm in diameter, 300 μg/mL) for 2 h. **(A)** The cell death rate was determined by measuring lactate dehydrogenase (LDH) activity in the culture supernatants. **(B,C)** Interleukin-1 alpha (IL-1α) and IL-1 beta (β) levels in the culture supernatants were measured using enzyme-linked immunosorbent assay (ELISA). **(D–F)** The primed BMDMs were treated with dasatinib (20 µM) and stimulated or not stimulated with SPs (1,500 nm in diameter, 300 μg/mL), yellow dust (500 μg/mL), monosodium urate (MSU) (300 μg/mL) or adenosine triphosphate (ATP) (3 mM) for 2 h. **(D)** The cell death rate was determined by measuring LDH activity in the culture supernatants. **(E,F)** IL-1α and IL-1β levels in the culture supernatants were measured using ELISA. The results are presented as the mean ± SD of values from triplicate wells. **, *p* < 0.01; ***, *p* < 0.001; and ****, *p* < 0.0001.

Particulates exhibit size-dependent variations in inflammatory properties and cytotoxicities (Kusaka et al., 2014; Nishijima et al., 2017; Chen et al., 2022). We evaluated the suppressive effects of dasatinib on pyroptosis induced by SPs of different sizes. An actin-polymerization inhibitor, Cyto D, almost completely suppressed cell death accompanied by IL-1α and IL-1β release in BMDMs induced by SPs with diameters of 500, 1,500, and 3,000 nm (Figures 3A–C), indicating these SPs were incorporated via phagocytosis. Thus, dasatinib significantly suppressed the cell death of BMDMs accompanied by IL-1α and IL-1β release induced by SPs, the efficacy of which varied with SP size.

Furthermore, dasatinib suppressed cell death and IL-1α and IL-1β release induced by yellow dust, which contains crystalline silica, and MSU crystals (Figures 3D–F), indicating that it suppresses pyroptosis induced by particulates of various materials.

In addition, we determined whether dasatinib suppresses pyroptosis induced by external stimuli other than particulates. ATP activates the NLRP3 inflammasome by inducing mitochondrial dysfunction, resulting in pyroptosis (Yang et al., 2019). However, dasatinib did not suppress ATP-induced cell death and IL-1α and IL-1β release (Figures 3D–F). Therefore, the suppressive effects of dasatinib on pyroptosis are selective and are strongly exhibited during particulate-induced pyroptosis.

### Dasatinib suppresses particulate-induced phagolysosomal dysfunction without affecting phagocytosis

**FIGURE 4:**
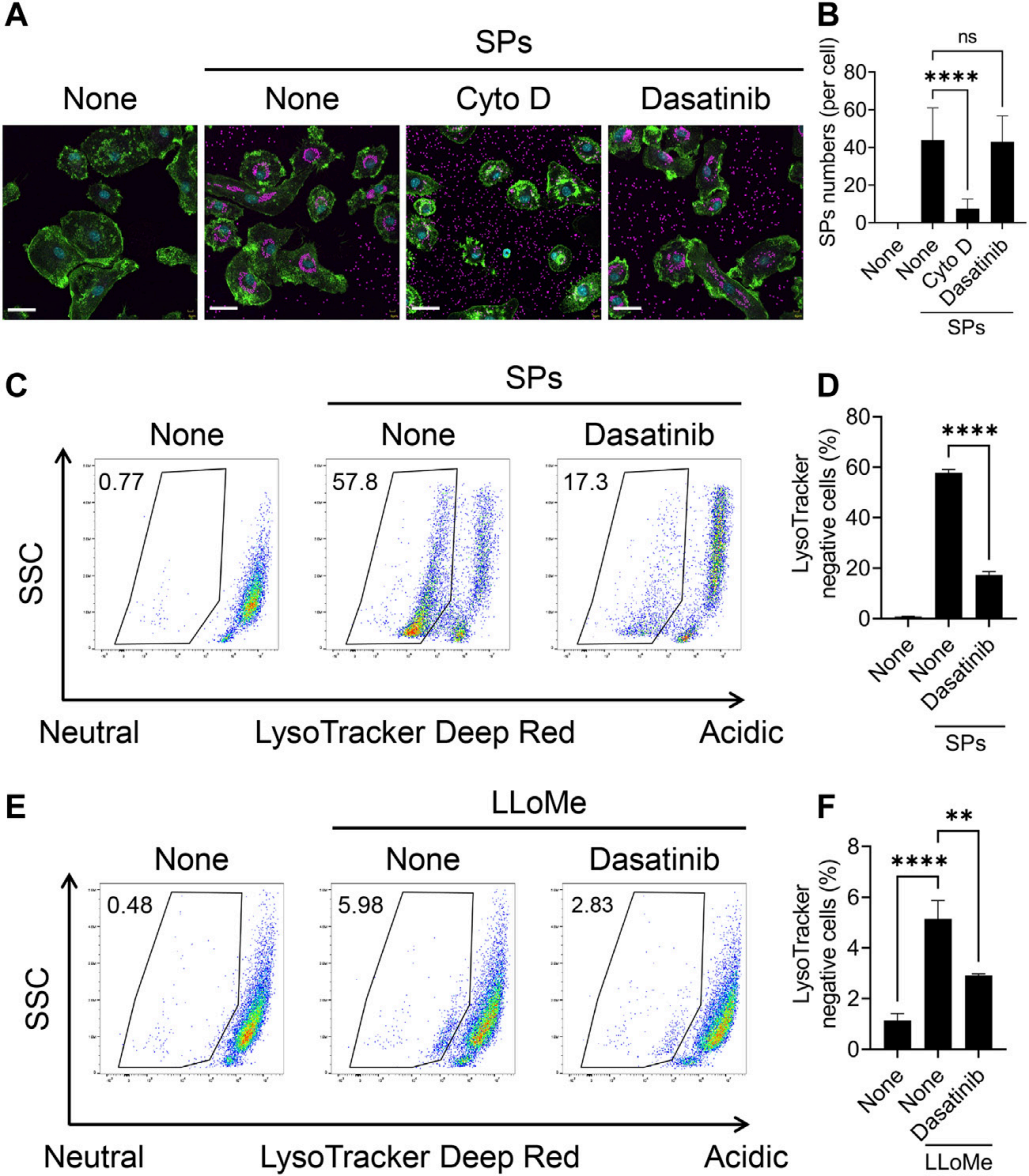
Dasatinib suppresses phagolysosomal and lysosomal dysfunction induced by silica particle (SP) and L-leucyl-L-leucine methyl ester (LLoMe) stimulation. **(A)** Primed bone marrow-derived macrophages (BMDMs) were treated with 20 µM cytochalasin D (Cyto D) or dasatinib and stimulated or not stimulated with fluorescent SPs (1,500 nm in diameter, 20 μg/mL; magenta) for 2 h. Actin (green) and nuclei (cyan) were stained with phalloidin and DRAQ5, respectively. Scale bar; 30 µm. **(B)** Numbers of SPs in BMDMs. The results are presented as the mean ± SD of values from 74 cells. **(C)** Flow cytometric analysis of the primed BMDMs treated with dasatinib (20 µM) and stimulated or not stimulated with SPs (1,500 nm in diameter, 300 μg/mL) for 2 h. The cells were stained with the fluorescent dye LysoTracker Deep Red. The data are representative of three independent experiments. **(D)** Percentages of the LysoTracker Deep Red-negative population were calculated. The results are presented as the mean ± SD of values from triplicate wells. **(E)** Flow cytometric analysis of primed BMDMs treated with dasatinib (20 µM) and stimulated or not stimulated with LLoMe (0.5 µM) for 3 h. The cells were stained with the fluorescent dye LysoTracker Deep Red. The data are representative of three independent experiments. **(F)** Percentages of the LysoTracker Deep Red-negative population were calculated. The results are presented as the mean ± SD of values from triplicate wells. **, *p* < 0.01, and ****, *p* < 0.0001; ns, not significant.

We further attempted to elucidate the mechanism underlying the effect of dasatinib on particulate-induced cellular responses. SFKs regulate Fcγ receptor-mediated phagocytosis (Suzuki et al., 2000). Therefore, we investigated whether dasatinib inhibits the phagocytosis of SPs by BMDMs. BMDMs were visualized by staining the nuclei and filamentous actin with fluorescent dyes after stimulation with fluorescent SPs and counting the number of SPs incorporated into the cytoplasm under a microscope (Figure 4A). Cyto D markedly decreased the number of incorporated SPs; whereas, dasatinib had no effects on SP phagocytosis (Figure 4B). Therefore, the suppression of particulate-induced pyroptosis by dasatinib is unlikely to be caused by the inhibition of phagocytic activity against particulates.

Flow cytometric analysis of BMDMs stained with LysoTracker Deep Red, an acidotropic fluorescent dye which labels acidic organelles, including phagolysosomes, was performed to elucidate whether dasatinib suppresses SP-induced phagolysosomal dysfunction (Ikoma et al., 2022). The stimulation of BMDMs with SPs decreased the fluorescence intensity of LysoTracker Deep Red, indicating loss of the interior acidity of the phagolysosomes owing to leakage of their contents (Figures 4C, D). Dasatinib significantly decreased the population of LysoTracker Deep Red-negative cells among SP-stimulated cells. Thus, dasatinib was found to suppress particulate-induced phagolysosomal dysfunction.

Furthermore, we elucidated whether dasatinib reduced lysosomal dysfunction and subsequent pyroptosis induced by the lysosomotropic compound LLoMe. LLoMe accumulates in the lysosomes and is processed by the lysosomal thiol protease dipeptidyl peptidase I (Uchimoto et al., 1999). This process results in lysosomal dysfunction. Dasatinib decreased the population of LysoTracker Deep Red-negative cells among LLoMe-stimulated cells (Figures 4E, F). Furthermore, it significantly suppressed LLoMe-induced cell death and IL-1α and IL-1β release (Supplementary Figures S3A–C). Therefore, these results indicating that dasatinib suppressed pyroptosis by preventing phagolysosomal and lysosomal dysfunction.

### Phosphorylated SFKs accumulate around particulate-engulfed phagosomes

**FIGURE 5:**
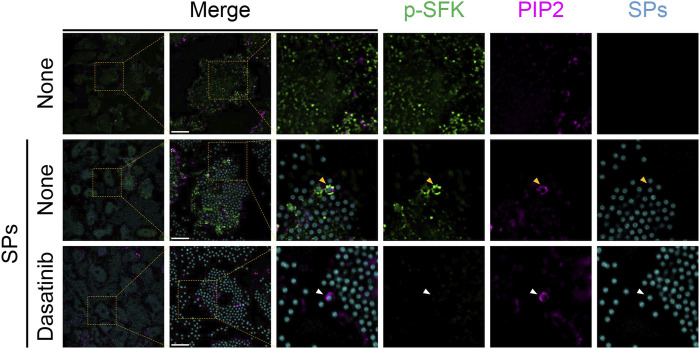
Phosphorylated Src family kinases (p-SFKs) accumulate around silica particle (SP)-containing phagocytic cups shortly after the phagocytosis of SPs. Primed bone marrow-derived macrophages (BMDMs) were treated with dasatinib (20 µM) and stimulated or not stimulated with fluorescent SPs (1,500 nm in diameter, 300 μg/mL; cyan). 15 min after stimulation with fluorescent SPs, p-SFKs (green) and phosphatidylinositol 4,5-bisphosphate (PIP2) (magenta) were stained with specific antibodies. The white arrowhead indicates SP surrounded by PIP2. Yellow arrowheads indicate SPs surrounded by both p-SFKs and PIP2. Scale bar; 10 µm.

Based on the findings of the present study, SFKs potentially mediate the dysfunction of SP-containing phagolysosomes. SFKs, including Src, Fyn, Lyn, and Yes, are recruited to actin-rich phagocytic cups during Fcγ receptor-mediated phagocytosis (Majeed et al., 2001). Therefore, we performed immunofluorescence analysis of BMDMs to investigate the spatio-temporal activity of SFK after the cells phagocytose particulates. In the initial stages of phagocytosis, PIP2 accumulates and initiates actin polymerization to extend the pseudopod and engulf the target (e.g., particulates) (Scott et al., 2005). Without SP stimulation, both PIP2 and p-SFKs were distributed throughout the cytoplasm of LPS-primed BMDMs (Figure 5). SPs were surrounded by PIP2 in BMDMs 15 min after SP stimulation. Furthermore, p-SFK accumulated around some PIP2-surrounded SPs. Dasatinib abolished p-SFK signals but did not affect the intensity or distribution of the PIP2 signals. These observations suggest that p-SFKs accumulate around the phagosomes engulfing particulates during the initial stages of phagocytosis.

**FIGURE 6:**
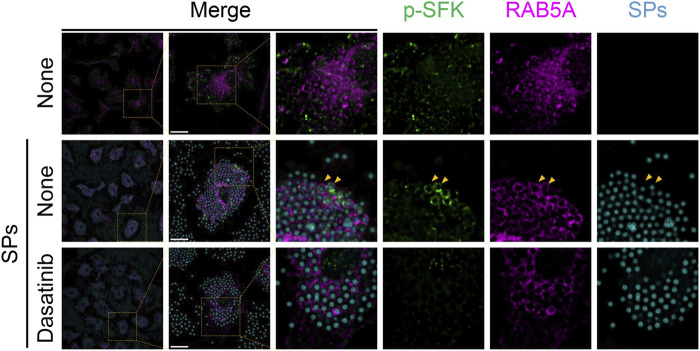
Phosphorylated Src family kinases (p-SFKs) accumulate around silica particle (SP)-containing early phagosomes. Primed bone marrow-derived macrophages (BMDMs) were treated with dasatinib (20 µM) and stimulated or not stimulated with fluorescent SPs (1,500 nm in diameter, 300 μg/mL; cyan). Thirty minutes after stimulation with fluorescent SPs, p-SFKs (green) and RAB5A (magenta) were stained with specific antibodies. Yellow arrowheads indicate SPs surrounded by both p-SFKs and RAB5A. Scale bar; 10 µm.

**FIGURE 7:**
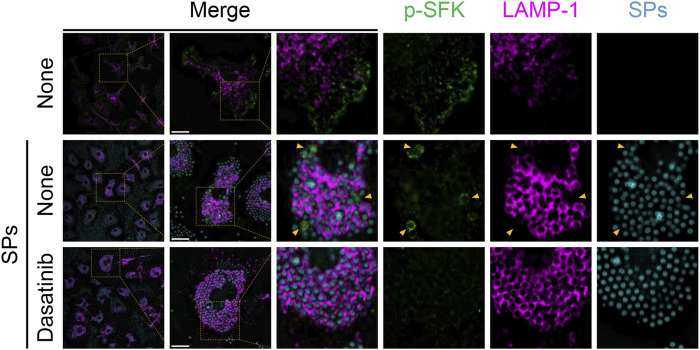
Silica particle (SP)-containing phagosomes are surrounded by phosphorylated Src family kinases (p-SFKs) but not lysosome-associated membrane protein-1 (LAMP-1). Primed bone marrow-derived macrophages (BMDMs) were treated with dasatinib (20 µM) and stimulated or not stimulated with fluorescent SPs (1,500 nm in diameter, 300 μg/mL; cyan). Thirty minutes after stimulation with fluorescent SPs, p-SFKs (green), and LAMP-1 (magenta) were stained with specific antibodies. Yellow arrowheads indicate p-SFK-surrounded SPs. Scale bar; 10 µm.

Next, we stained p-SFKs with an early phagosome marker, RAB5A, 30 min after SP stimulation. We observed RAB5A-positive vesicles in the cytoplasm of BMDMs without SP stimulation (Figure 6). Most of the engulfed SPs were surrounded by RAB5A, and some of the RAB5A-surrounded SPs were also surrounded by p-SFK. Dasatinib did not influence the intensity or distribution of RAB5A signals. Furthermore, we performed immunostaining for LAMP-1, a lysosomal membrane protein used as a marker for late phagosomes and phagolysosomes (Kinchen and Ravichandran, 2008). We detected LAMP-1-positive vesicles in the cytoplasm of BMDMs without SP stimulation (Figure 7). Thirty minutes after stimulation, SPs were surrounded by LAMP-1, indicating that SP-engulfed phagosomes matured on fusing with lysosomes. However, LAMP-1 was not detected around p-SFK-surrounded SPs. Dasatinib did not affect LAMP-1 distribution after SP stimulation. In summary, p-SFKs may act on particulate-engulfed phagosomes mainly during the early stages of maturation.

### Dasatinib treatment alleviates particulate-induced lung inflammation

**FIGURE 8:**
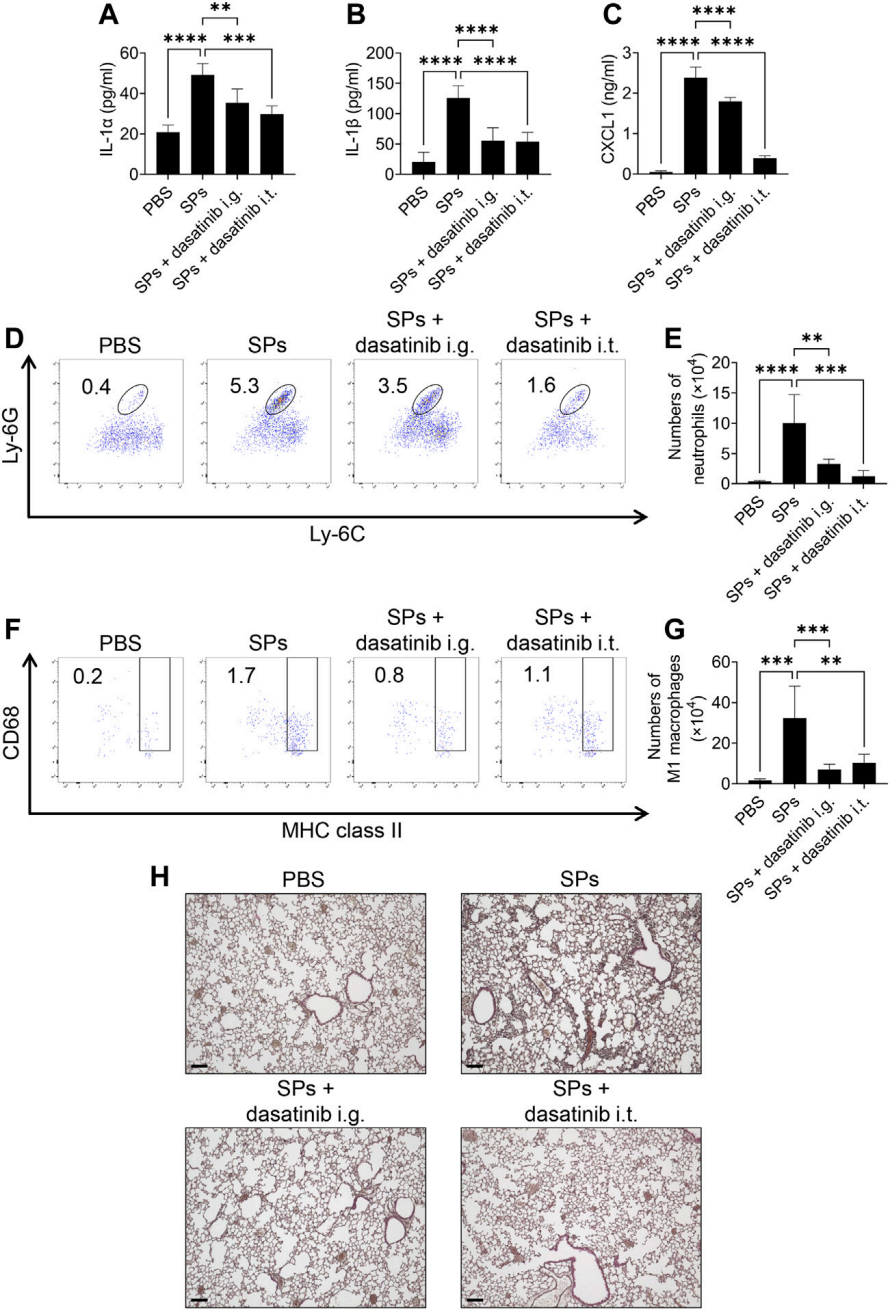
Dasatinib treatment attenuates SP-induced acute pneumonia. **(A–C)** Bronchoalveolar lavage (BAL) fluid was collected from mice 12 h after intratracheal SP administration (i.t.; 100 mg/kg) with or without the intragastric (i.g.; 30 mg/kg) and intratracheal (10 mg/kg) administration of dasatinib. Interleukin-1 alpha (IL-1α), IL-1 beta (β), and chemokine (C-X-C motif) ligand 1 (CXCL1) levels in the BAL fluid were measured using enzyme-linked immunosorbent assay (ELISA). **(D)** Neutrophils in mouse lungs 12 h after intratracheal SP administration were counted using flow cytometry. The numbers in the plots indicate the percentage of neutrophils in total leukocytes. **(E)** The total number of neutrophils. **(F)** M1 macrophages in mouse lungs 12 h after intratracheal SP administration were counted using flow cytometry. The numbers in the plots indicate the percentage of M1 macrophages in total leukocytes. **(G)** The total number of M1 macrophages. **(H)** Representative images of hematoxylin and eosin staining of mouse lungs collected 12 h after intratracheal SP administration. Scale bar; 100 μm. The results are presented as mean ± SD (*n* = 5, each group). **, *p* < 0.01; ***, *p* < 0.001; and ****, *p* < 0.0001.

Finally, we evaluated the anti-inflammatory effects of dasatinib on SP-induced lung inflammation in mice. In this model, intratracheally administered SPs induce IL-1α release from pulmonary macrophages in an NLRP3-independent manner, resulting in neutrophil recruitment and inflammation (Ikoma et al., 2022). Dasatinib is an orally administered drug. We also tested topical treatment of the lung with dasatinib by its intratracheal administration. Both, intragastric and intratracheal administrations of dasatinib suppressed SP-induced lung inflammation. Dasatinib administrations via these routes significantly decreased IL-1α and IL-1β levels in the BAL fluid of mice after the intratracheal administration of SPs (Figures 8A, B). Furthermore, dasatinib administration through both the routes suppressed the levels of a neutrophil chemoattractant, CXCL1, and decreased the number of neutrophils in the mouse lungs after SP administration, although the inhibitory effect was stronger with intratracheal administration than that with intragastric administration (Figures 8C–E). These routes also suppressed the increase in pro-inflammatory M1 macrophages in the mouse lungs after SP administration (Figures 8F, G). Consistent with these findings, histological analysis revealed that both dasatinib treatment routes suppressed immune cell infiltration into the lungs after SP administration (Figure 8H). In summary, dasatinib effectively suppressed particulate-induced NLRP3-independent lung inflammation.

## Discussion

To our knowledge, this study is the first to demonstrate that the FDA-approved drug, dasatinib, suppresses particulate-induced cell death and IL-1α release, both of which occur even in the absence of the NLRP3 inflammasome (Ikoma et al., 2022). The results suggest that dasatinib decreased particulate-induced phagolysosomal dysfunction, thereby preventing pyroptosis. A previous study reported that c-Src^Y527F^ (a constitutively active form of c-Src)-transduced murine embryonic fibroblasts become increasingly sensitive to drug-induced cell death, triggering permeabilization of the lysosomal membrane (Fehrenbacher et al., 2008). In addition, c-Src inhibition decreases *Mycobacterium tuberculosis*-induced lysosomal destabilization (Amaral et al., 2018). Therefore, dasatinib-targeted SFKs may promote particulate-induced phagolysosomal membrane destabilization. Interestingly, p-SFKs accumulated around the phagocytic cups formed immediately after the phagocytosis of particulates and persisted even after becoming early phagosomes. SFKs activate acid sphingomyelinase (ASM), a lysosomal ceramide-producing enzyme (Kumazoe et al., 2020), which is present in early phagosomes (Wähe et al., 2010). When activated, ASM converts membrane sphingomyelin to ceramide, which is then converted to sphingosine by ceramidase (Gault et al., 2010). The accumulation of sphingosine enhances membrane permeabilization in a detergent-like manner (Boya and Kroemer, 2008). Interestingly, ASM inhibitors inhibit SP-induced cell death (Thibodeau et al., 2004; Biswas et al., 2017). Therefore, ASM may function as a downstream factor of dasatinib-targeted SFKs to facilitate the membrane destabilization of phagosomes and/or phagolysosomes that engulf particulates. Furthermore, dasatinib-targeted SFKs may act on lysosomal ASM, thereby enhancing destabilization of the lysosomal membrane induced by non-particulate agents, such as LLoMe.

In the present study, p-SFKs accumulated around SPs surrounded by RAB5A. However, LAMP-1 levels around SPs surrounded by p-SFKs were negligible. Therefore, it is presumed that dasatinib-targeted SFKs act on the membranes of particulate-containing phagosomes only at the early stage of maturation; however, whether p-SFKs are dispersed, dephosphorylated, or degraded after the maturation of phagosomes into phagolysosomes remains unclear. Alternatively, p-SFK-accumulated phagosomes are vulnerable and immediately degrade after fusion with lysosomes. In our study, p-SFK-accumulated phagolysosomes were not detected under the experimental conditions. Therefore, further studies are warranted on p-SFK recruitment after engulfing particulates and their subsequent fate. These studies will help explain why p-SFKs are selectively recruited to certain phagosomes.

Microbial components adhere to the surfaces of externally invading particulates (Reed and Milton, 2001; Ichinose et al., 2008; He et al., 2013). The stimulation of pattern recognition receptors by microbial components, including toll-like receptor 4, can induce an increase in and activate dasatinib-targeted SFKs. In addition, pattern recognition receptor activation induces the expression of inflammatory mediators, including IL-1α. These effects may synergistically elevate the inflammatory responses triggered by pyroptosis on exposure to exogenously generated particulates. We also demonstrated that LPS priming enhanced SP-induced cell death and IL-1α release. Importantly, SFK inhibition by dasatinib strongly suppressed SP-induced pyroptosis, including enhancement by LPS priming. Our study results suggest that dasatinib-targeted SFKs mediate particulate-induced phagolysosomal dysfunction, an early upstream event resulting in cell death; therefore, they are critical in determining the intensity of inflammation associated with particulate-induced pyroptosis. As a result, SFK inhibition could be an effective and rational approach to prevent excessive inflammation caused by particulates. This approach could be applicable for treating various particulate-induced diseases since dasatinib suppresses pyroptosis induced by particulates of various sizes and materials.

Moreover, we demonstrated that dasatinib treatment effectively ameliorated inflammatory manifestations in SP-induced acute lung injury in mice, characterized by the increased levels of IL-1α, IL-1β, CXCL1, neutrophils, and M1 macrophages. Importantly, the clinically relevant intragastric administration of dasatinib remarkably suppressed the SP-induced pulmonary inflammation and was as effective as its pulmonary administration. Neutrophils play an important role in acute lung injury after the inhalation of particulate irritants (Ikoma et al., 2022). Therefore, the oral administration of dasatinib can be used as an emergency measure to alleviate acute lung injury induced by the inhalation of particulate irritants. In general, SFKs are multifunctional enzymes; therefore, the use of SFK inhibitors, such as dasatinib, in clinical settings is currently limited to treating cancers, such as chronic myeloid leukemia (Aguilera and Tsimberidou, 2009). The inhalation of particulate irritants in humans causes pneumoconiosis, characterized by persistent inflammation often accompanied by lung cancer (Tsuda et al., 1997; Rayens et al., 2022). However, only palliative treatment exists for pneumoconiosis. IL-1α may promote the development of lung cancer (Chiu et al., 2021). Furthermore, neutrophilic inflammation is involved in silica-induced lung cancer progression (Satpathy et al., 2015). To expand the range of treatments with SFK inhibitors, including dasatinib, their application for treating pneumoconiosis-associated lung cancer may be promising, particularly in the context of this study. In addition, chronic inflammation associated with pneumoconiosis often causes fibrosis. Dasatinib treatment is known to be effective in suppressing SP-induced pulmonary fibrosis (Cruz et al., 2016). Considering that IL-1 is involved in the development of SP-induced pulmonary fibrosis (Piguet et al., 1993), the suppression of pyroptosis by dasatinib may contribute to its anti-fibrotic efficacy. The continuous intake of SFK inhibitors would be required to suppress chronic inflammation associated with pneumoconiosis and prevent lung carcinogenesis and fibrosis. In future, identifying SFKs that destabilize phagolysosomal membranes and their target molecules will aid in the development of more efficient, specific, and safe drugs for particulate-induced inflammatory disease.

## Data Availability

The original contributions presented in the study are included in the article/Supplementary Material, further inquiries can be directed to the corresponding authors.

## Abbreviations

ASM, acid sphingomyelinase; ATP, adenosine triphosphate; BAL, bronchoalveolar lavage; BMDMs, bone marrow-derived macrophages; CXCL, chemokine (C-X-C motif) ligand; Cyto D, cytochalasin D; ELISA, enzyme-linked immunosorbent assay; FCS, fetal calf serum; FDA, Food and Drug Administration; HRP, horseradish peroxidase; IL, interleukin; LAMP-1, lysosome-associated membrane protein-1; LDH, lactate dehydrogenase; LLoMe, L-leucyl-L-leucine methyl ester; LPS, lipopolysaccharide; MSU, monosodium urate; NLRP3, Nod-like receptor family pyrin domain containing 3; PIP2, phosphatidylinositol 4,5-bisphosphate; p-SFK, phosphorylated Src family kinase; SFK, Src family kinase; SP, silica particle.

## References

[B1] Aguilera D. G. Tsimberidou A. M. (2009). Dasatinib in chronic myeloid leukemia: a review. Ther. Clin. Risk Manag. 5, 281–289. 10.2147/tcrm.s3425 19536317 PMC2697539

[B2] Amaral E. P. Riteau N. Moayeri M. Maier N. Mayer-Barber K. D. Pereira R. M. (2018). Lysosomal cathepsin release is required for NLRP3-inflammasome activation by *Mycobacterium tuberculosis* in infected macrophages. Front. Immunol. 9, 1427. 10.3389/fimmu.2018.01427 29977244 PMC6021483

[B3] Araujo J. Logothetis C. (2010). Dasatinib: a potent SRC inhibitor in clinical development for the treatment of solid tumors. Cancer Treat. Rev. 36, 492–500. 10.1016/j.ctrv.2010.02.015 20226597 PMC3940067

[B4] Barquera S. Pedroza-Tobías A. Medina C. Hernández-Barrera L. Bibbins-Domingo K. Lozano R. (2015). Global overview of the epidemiology of atherosclerotic cardiovascular disease. Arch. Med. Res. 46, 328–338. 10.1016/j.arcmed.2015.06.006 26135634

[B5] Biswas R. Trout K. L. Jessop F. Harkema J. R. Holian A. (2017). Imipramine blocks acute silicosis in a mouse model. Part. Fibre. Toxicol. 14, 36. 10.1186/s12989-017-0217-1 28893276 PMC5594487

[B6] Boggon T. J. Eck M. J. (2004). Structure and regulation of Src family kinases. Oncogene 23, 7918–7927. 10.1038/sj.onc.1208081 15489910

[B7] Boya P. Kroemer G. (2008). Lysosomal membrane permeabilization in cell death. Oncogene 27, 6434–6451. 10.1038/onc.2008.310 18955971

[B8] Byeon S. E. Yi Y.-S. Oh J. Yoo B. C. Hong S. Cho J. Y. (2012). The role of Src kinase in macrophage-mediated inflammatory responses. Mediat. Inflamm. 2012, 512926. 10.1155/2012/512926 PMC3504478 23209344

[B9] Chen C. Wang J. Liang Z. Li M. Fu D. Zhang L. (2022). Monosodium urate crystals with controlled shape and aspect ratio for elucidating the pathological progress of acute gout. Biomater. Adv. 139, 213005. 10.1016/j.bioadv.2022.213005 35882152

[B10] Chiu J. W. Binte Hanafi Z. Chew L. C. Y. Mei Y. Liu H. (2021). IL-1α processing, signaling and its role in cancer progression. Cells 10, 92. 10.3390/cells10010092 33430381 PMC7827341

[B11] Cho W.-S. Choi M. Han B. S. Cho M. Oh J. Park K. (2007). Inflammatory mediators induced by intratracheal instillation of ultrafine amorphous silica particles. Toxicol. Lett. 175, 24–33. 10.1016/j.toxlet.2007.09.008 17981407

[B12] Cruz F. F. Horta L. F. Maia Lde A. Lopes-Pacheco M. da Silva A. B. Morales M. M. (2016). Dasatinib reduces lung inflammation and fibrosis in acute experimental silicosis. PLoS One 11, e0147005. 10.1371/journal.pone.0147005 26789403 PMC4720427

[B13] Dorsey J. Cunnick J. Lanehart R. Huang M. Kraker A. Bhalla K. (2002). Interleukin-3 protects Bcr-Abl-transformed hematopoietic progenitor cells from apoptosis induced by Bcr-Abl tyrosine kinase inhibitors. Leukemia 16, 1589–1595. 10.1038/sj.leu.2402678 12200668

[B14] Dostert C. Pétrilli V. Van Bruggen R. Steele C. Mossman B. T. Tschopp J. r. (2008). Innate immune activation through Nalp3 inflammasome sensing of asbestos and silica. Science 320, 674–677. 10.1126/science.1156995 18403674 PMC2396588

[B15] Duewell P. Kono H. Rayner K. J. Sirois C. M. Vladimer G. Bauernfeind F. G. (2010). NLRP3 inflammasomes are required for atherogenesis and activated by cholesterol crystals. Nature 464, 1357–1361. 10.1038/nature08938 20428172 PMC2946640

[B16] Fehrenbacher N. Bastholm L. Kirkegaard-Sørensen T. Rafn B. Bøttzauw T. Nielsen C. (2008). Sensitization to the lysosomal cell death pathway by oncogene-induced down-regulation of lysosome-associated membrane proteins 1 and 2. Cancer Res. 68, 6623–6633. 10.1158/0008-5472.CAN-08-0463 18701486

[B17] Gault C. R. Obeid L. M. Hannun Y. A. (2010). An overview of sphingolipid metabolism: from synthesis to breakdown. Adv. Exp. Med. Biol. 688, 1–23. 10.1007/978-1-4419-6741-1_1 20919643 PMC3069696

[B18] Golas J. M. Arndt K. Etienne C. Lucas J. Nardin D. Gibbons J. (2003). SKI-606, a 4-anilino-3-quinolinecarbonitrile dual inhibitor of Src and Abl kinases, is a potent antiproliferative agent against chronic myelogenous leukemia cells in culture and causes regression of K562 xenografts in nude mice. Cancer Res. 63, 375–381. 12543790

[B19] Groß O. Yazdi A. S. Thomas C. J. Masin M. Heinz L. X. Guarda G. (2012). Inflammasome activators induce interleukin-1α secretion via distinct pathways with differential requirement for the protease function of caspase-1. Immunity 36, 388–400. 10.1016/j.immuni.2012.01.018 22444631

[B20] Halle A. Hornung V. Petzold G. C. Stewart C. R. Monks B. G. Reinheckel T. (2008). The NALP3 inflammasome is involved in the innate immune response to amyloid-beta. Nat. Immunol. 9, 857–865. 10.1038/ni.1636 18604209 PMC3101478

[B21] Haloul M. Oliveira E. R. A. Kader M. Wells J. Z. Tominello T. R. El Andaloussi A. (2019). mTORC1-mediated polarization of M1 macrophages and their accumulation in the liver correlate with immunopathology in fatal ehrlichiosis. Sci. Rep. 9, 14050. 10.1038/s41598-019-50320-y 31575880 PMC6773708

[B22] Hamby J. M. Connolly C. J. Schroeder M. C. Winters R. T. Showalter H. D. Panek R. L. (1997). Structure-activity relationships for a novel series of pyrido[2,3-d]pyrimidine tyrosine kinase inhibitors. J. Med. Chem. 40, 2296–2303. 10.1021/jm970367n 9240345

[B23] He M. Ichinose T. Song Y. Yoshida Y. Arashidani K. Yoshida S. (2013). Effects of two Asian sand dusts transported from the dust source regions of Inner Mongolia and northeast China on murine lung eosinophilia. Toxicol. Appl. Pharmacol. 272, 647–655. 10.1016/j.taap.2013.07.010 23896513

[B24] Hornung V. Bauernfeind F. Halle A. Samstad E. O. Kono H. Rock K. L. (2008). Silica crystals and aluminum salts activate the NALP3 inflammasome through phagosomal destabilization. Nat. Immunol. 9, 847–856. 10.1038/ni.1631 18604214 PMC2834784

[B25] Ichinose T. Yoshida S. Hiyoshi K. Sadakane K. Takano H. Nishikawa M. (2008). The effects of microbial materials adhered to Asian sand dust on allergic lung inflammation. Arch. Environ. Contam. Toxicol. 55, 348–357. 10.1007/s00244-007-9128-8 18227959

[B26] Ikoma K. Takahama M. Kimishima A. Pan Y. Taura M. Nakayama A. (2022). Oridonin suppresses particulate-induced NLRP3-independent IL-1α release to prevent crystallopathy in the lung. Int. Immunol. 34, 493–504. 10.1093/intimm/dxac018 35639943

[B27] Kayagaki N. Kornfeld O. S. Lee B. L. Stowe I. B. O'Rourke K. Li Q. (2021). NINJ1 mediates plasma membrane rupture during lytic cell death. Nature 591, 131–136. 10.1038/s41586-021-03218-7 33472215

[B28] Kimura Y. Tsukui D. Kono H. (2021). Uric acid in inflammation and the pathogenesis of atherosclerosis. Int. J. Mol. Sci. 22, 12394. 10.3390/ijms222212394 34830282 PMC8624633

[B29] Kinchen J. M. Ravichandran K. S. (2008). Phagosome maturation: going through the acid test. Nat. Rev. Mol. Cell Biol. 9, 781–795. 10.1038/nrm2515 18813294 PMC2908392

[B30] Kumazoe M. Kadomatsu M. Bae J. Otsuka Y. Fujimura Y. Tachibana H. (2020). Src mediates epigallocatechin-3-O-Gallate-Elicited acid sphingomyelinase activation. Molecules 25, 5481. 10.3390/molecules25225481 33238540 PMC7700551

[B31] Kuroda E. Ozasa K. Temizoz B. Ohata K. Koo C. X. Kanuma T. (2016). Inhaled fine particles induce alveolar macrophage death and interleukin-1α release to promote inducible bronchus-associated lymphoid tissue formation. Immunity 45, 1299–1310. 10.1016/j.immuni.2016.11.010 28002730

[B32] Kusaka T. Nakayama M. Nakamura K. Ishimiya M. Furusawa E. Ogasawara K. (2014). Effect of silica particle size on macrophage inflammatory responses. PloS one 9, e92634. 10.1371/journal.pone.0092634 24681489 PMC3969333

[B33] Lombardo L. J. Lee F. Y. Chen P. Norris D. Barrish J. C. Behnia K. (2004). Discovery of N-(2-chloro-6-methyl- phenyl)-2-(6-(4-(2-hydroxyethyl)- piperazin-1-yl)-2-methylpyrimidin-4- ylamino)thiazole-5-carboxamide (BMS-354825), a dual Src/Abl kinase inhibitor with potent antitumor activity in preclinical assays. J. Med. Chem. 47, 6658–6661. 10.1021/jm049486a 15615512

[B34] Lopez O. L. Kuller L. H. (2019). Epidemiology of aging and associated cognitive disorders: prevalence and incidence of Alzheimer's disease and other dementias. Handb. Clin. Neurol. 167, 139–148. 10.1016/b978-0-12-804766-8.00009-1 31753130

[B35] Maa M. C. Leu T. H. (2016). Src is required for migration, phagocytosis, and interferon beta production in Toll-like receptor-engaged macrophages. Biomed. (Taipei) 6, 14. 10.7603/s40681-016-0014-4 PMC4980824 27514533

[B36] Majeed M. Caveggion E. Lowell C. A. Berton G. (2001). Role of Src kinases and Syk in Fcγ receptor-mediated phagocytosis and phagosome-lysosome fusion. J. Leukoc. Biol. 70, 801–811. 10.1189/jlb.70.5.801 11698501

[B37] Martinon F. Pétrilli V. Mayor A. Tardivel A. Tschopp J. (2006). Gout-associated uric acid crystals activate the NALP3 inflammasome. Nature 440, 237–241. 10.1038/nature04516 16407889

[B38] Matsui Y. Takemura N. Shirasaki Y. Takahama M. Noguchi Y. Ikoma K. (2022). Nanaomycin E inhibits NLRP3 inflammasome activation by preventing mitochondrial dysfunction. Int. Immunol. 34, 505–518. 10.1093/intimm/dxac028 35759801

[B39] Mu Y. Sun J. Li Z. Zhang W. Liu Z. Li C. (2022). Activation of pyroptosis and ferroptosis is involved in the hepatotoxicity induced by polystyrene microplastics in mice. Chemosphere 291, 132944. 10.1016/j.chemosphere.2021.132944 34793849

[B40] Mulay S. R. Kulkarni O. P. Rupanagudi K. V. Migliorini A. Darisipudi M. N. Vilaysane A. (2012). Calcium oxalate crystals induce renal inflammation by NLRP3-mediated IL-1β secretion. J. Clin. Invest. 123, 236–246. 10.1172/JCI63679 23221343 PMC3533282

[B41] Nascimento Da Conceicao V. Sun Y. Ramachandran K. Chauhan A. Raveendran A. Venkatesan M. (2021). Resolving macrophage polarization through distinct Ca(2+) entry channel that maintains intracellular signaling and mitochondrial bioenergetics. iScience 24, 103339. 10.1016/j.isci.2021.103339 34816101 PMC8591423

[B42] Nishijima N. Hirai T. Misato K. Aoyama M. Kuroda E. Ishii K. J. (2017). Human scavenger receptor A1-mediated inflammatory response to silica particle exposure is size specific. Front. Immunol. 8, 379. 10.3389/fimmu.2017.00379 28421077 PMC5377922

[B43] Palmer B. D. Thompson A. M. Booth R. J. Dobrusin E. M. Kraker A. J. Lee H. H. (2006). 4-Phenylpyrrolo[3,4-c]carbazole-1,3(2H,6H)-dione inhibitors of the checkpoint kinase Wee1. Structure-activity relationships for chromophore modification and phenyl ring substitution. J. Med. Chem. 49, 4896–4911. 10.1021/jm0512591 16884302

[B44] Palomaki J. Valimaki E. Sund J. Vippola M. Clausen P. A. Jensen K. A. (2011). Long, needle-like carbon nanotubes and asbestos activate the NLRP3 inflammasome through a similar mechanism. ACS Nano 5, 6861–6870. 10.1021/nn200595c 21800904

[B45] Panek R. L. Lu G. H. Klutchko S. R. Batley B. L. Dahring T. K. Hamby J. M. (1997). *In vitro* pharmacological characterization of PD 166285, a new nanomolar potent and broadly active protein tyrosine kinase inhibitor. J. Pharmacol. Exp. Ther. 283, 1433–1444. 9400019

[B46] Parsons S. J. Parsons J. T. (2004). Src family kinases, key regulators of signal transduction. Oncogene 23, 7906–7909. 10.1038/sj.onc.1208160 15489908

[B47] Pelegrin P. Barroso-Gutierrez C. Surprenant A. (2008). P2X7 receptor differentially couples to distinct release pathways for IL-1beta in mouse macrophage. J. Immunol. 180, 7147–7157. 10.4049/jimmunol.180.11.7147 18490713

[B48] Piguet P. F. Vesin C. Grau G. E. Thompson R. C. (1993). Interleukin 1 receptor antagonist (IL-1ra) prevents or cures pulmonary fibrosis elicited in mice by bleomycin or silica. Cytokine 5, 57–61. 10.1016/1043-4666(93)90024-y 7683505

[B49] Rabolli V. Badissi A. A. Devosse R. Uwambayinema F. Yakoub Y. Palmai-Pallag M. (2014). The alarmin IL-1α is a master cytokine in acute lung inflammation induced by silica micro- and nanoparticles. Part. Fibre Toxicol. 11, 69. 10.1186/s12989-014-0069-x 25497724 PMC4279463

[B50] Rashidi M. Simpson D. S. Hempel A. Frank D. Petrie E. Vince A. (2019). The pyroptotic cell death effector gasdermin D is activated by gout-associated uric acid crystals but is dispensable for cell death and IL-1β release. J. Immunol. 203, 736–748. 10.4049/jimmunol.1900228 31209100 PMC6650356

[B51] Rayens N. T. Rayens E. A. Tighe R. M. (2022). Co-occurrence of pneumoconiosis with COPD, pneumonia and lung cancer. Occup. Med. (Lond). 72, 527–533. 10.1093/occmed/kqac079 35932472

[B52] Reed C. E. Milton D. K. (2001). Endotoxin-stimulated innate immunity: a contributing factor for asthma. J. Allergy Clin. Immunol. 108, 157–166. 10.1067/mai.2001.116862 11496229

[B53] Satpathy S. R. Jala V. R. Bodduluri S. R. Krishnan E. Hegde B. Hoyle G. W. (2015). Crystalline silica-induced leukotriene B4-dependent inflammation promotes lung tumour growth. Nat. Commun. 6, 7064. 10.1038/ncomms8064 25923988 PMC4418220

[B54] Scott C. C. Dobson W. Botelho R. J. Coady-Osberg N. Chavrier P. Knecht D. A. (2005). Phosphatidylinositol-4, 5-bis phosphate hydrolysis directs actin remodeling during phagocytosis. J. Cell Biol. 169, 139–149. 10.1083/jcb.200412162 15809313 PMC2171893

[B55] Suzuki T. Kono H. Hirose N. Okada M. Yamamoto T. Yamamoto K. (2000). Differential involvement of Src family kinases in Fcγ receptor-mediated phagocytosis. J. Immunol. 165, 473–482. 10.4049/jimmunol.165.1.473 10861086

[B56] Takeuchi O. Akira S. (2010). Pattern recognition receptors and inflammation. Cell 140, 805–820. 10.1016/j.cell.2010.01.022 20303872

[B57] Thibodeau M. S. Giardina C. Knecht D. A. Helble J. Hubbard A. K. (2004). Silica-induced apoptosis in mouse alveolar macrophages is initiated by lysosomal enzyme activity. Toxicol. Sci. 80, 34–48. 10.1093/toxsci/kfh121 15056807

[B58] Tsuda T. Babazono A. Yamamoto E. Mino Y. Matsuoka H. (1997). A meta-analysis on the relationship between pneumoconiosis and lung cancer. J. Occup. Health. 39, 285–294. 10.1539/joh.39.285

[B59] Uchimoto T. Nohara H. Kamehara R. Iwamura M. Watanabe N. Kobayashi Y. (1999). Mechanism of apoptosis induced by a lysosomotropic agent, L-Leucyl-L-Leucine methyl ester. Apoptosis 4, 357–362. 10.1023/a:1009695221038 14634338

[B60] von Schneidemesser E. Driscoll C. Rieder H. E. Schiferl L. D. (2020). How will air quality effects on human health, crops and ecosystems change in the future? Philos. Trans. A Math. Phys. Eng. Sci. 378, 20190330. 10.1098/rsta.2019.0330 32981439 PMC7536027

[B61] Wähe A. Kasmapour B. Schmaderer C. Liebl D. Sandhoff K. Nykjaer A. (2010). Golgi-to-phagosome transport of acid sphingomyelinase and prosaposin is mediated by sortilin. J. Cell Sci. 123, 2502–2511. 10.1242/jcs.067686 20571055

[B62] Yang Y. Wang H. Kouadir M. Song H. Shi F. (2019). Recent advances in the mechanisms of NLRP3 inflammasome activation and its inhibitors. Cell Death Dis. 10, 128. 10.1038/s41419-019-1413-8 30755589 PMC6372664

[B63] Yazdi A. S. Guarda G. Riteau N. Drexler S. K. Tardivel A. Couillin I. (2010). Nanoparticles activate the NLR pyrin domain containing 3 (Nlrp3) inflammasome and cause pulmonary inflammation through release of IL-1α and IL-1β. Proc. Natl. Acad. Sci. U. S. A. 107, 19449–19454. 10.1073/pnas.1008155107 20974980 PMC2984140

