# Supplementary material for: Dasatinib suppresses particulate-induced pyroptosis and acute lung inflammation
Source: Front Pharmacol. 2023 Aug 29;14:1250383. doi: 10.3389/fphar.2023.1250383 (PMC10495768; doi:10.3389/fphar.2023.1250383)
Supplement: Supplementary file 1 [file DataSheet1.PDF]

SUPPLEMENTARY FIGURE 1. Pan *et al.*

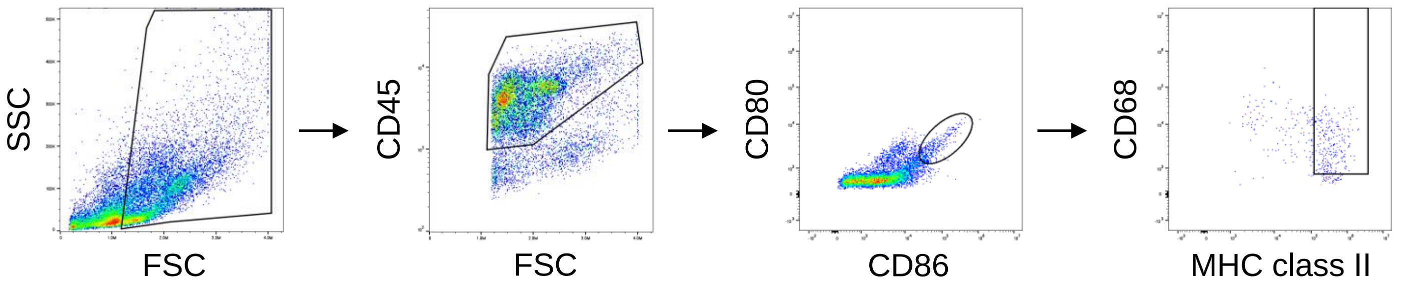

SUPPLEMENTARY FIGURE 1. Flow cytometry gating strategy used to define M1 macrophages. Leukocytes were gated based on forward scatter (FSC) versus side scatter (SSC) and then selected based the expression of CD45. CD86$^+$, CD80$^+$, major histocompatibility complex class II^{high}, CD68$^+$ cells were identified as M1 macrophages.

SUPPLEMENTARY FIGURE 2. Pan *et al.*

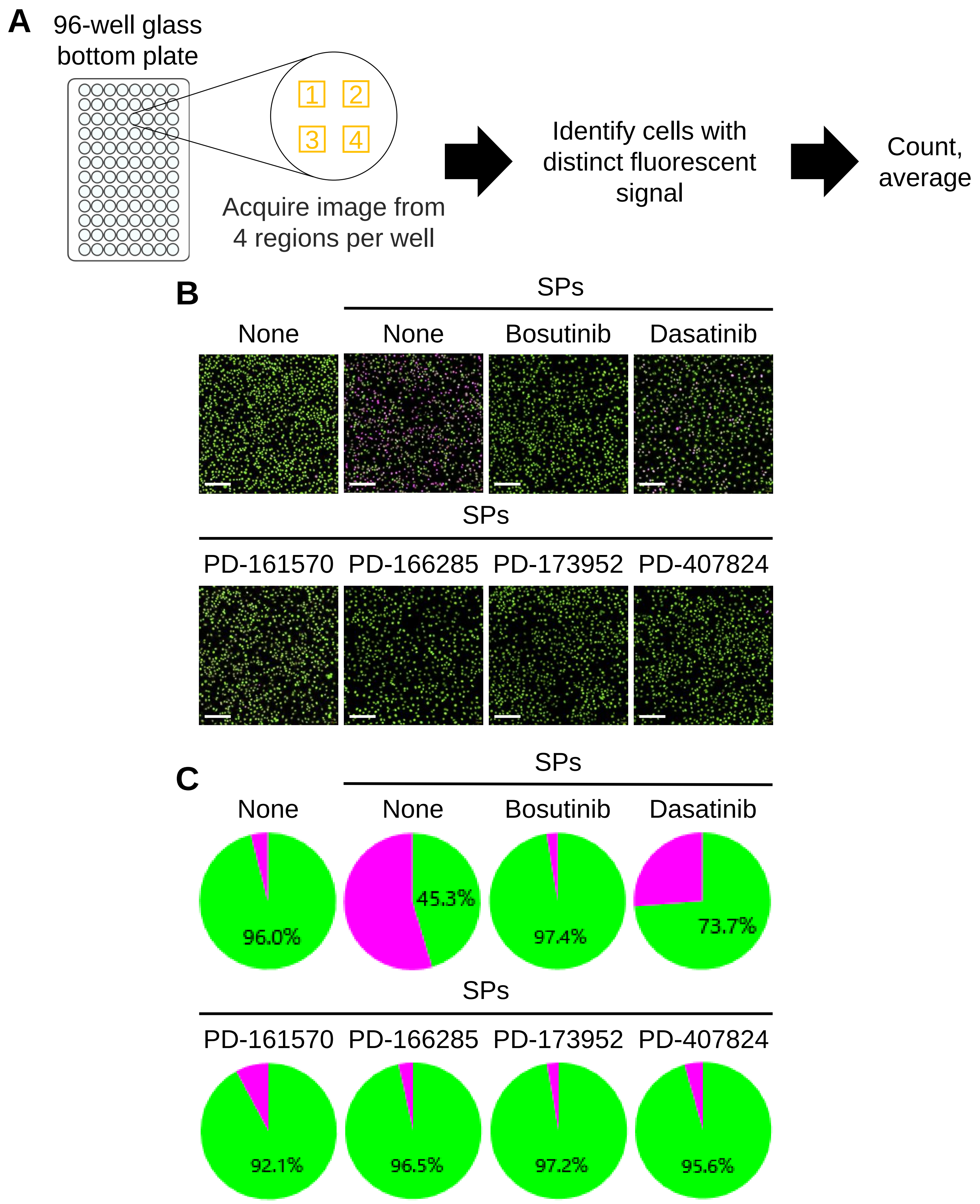

SUPPLEMENTARY FIGURE 2. Pan *et al.*

SUPPLEMENTARY FIGURE 2. Imaging-based high-throughput screening for compounds that inhibit SP-induced cell death. **(A)** The analysis procedure for the screening using CV8000. LPS-primed Raw264.7 cells were treated by each test compound (5 μM) from Pfizer drug library and FDA-approved drug library for 30 min and then stimulated with SPs (500 nm in diameter, 500 μg/ml), in the presence of Hoechst 33342 (1 μg/ml, green) and DRAQ7 (2 μM, magenta) for 2 h. The images were acquired at four different fields in each well. Hoechst 33342-positive, DRAQ7-negative cells and Hoechst 33342 and DRAQ7 double-positive cells were regarded as viable cells and dead cells, respectively. **(B)** The representative images of the cells treated with hit compounds. Scale bar, 120 μm. **(C)** The rate of viable cells (green) and dead cells (magenta) of **(B)** was calculated by CellPathfinder software.

SUPPLEMENTARY FIGURE 3. Pan *et al.*

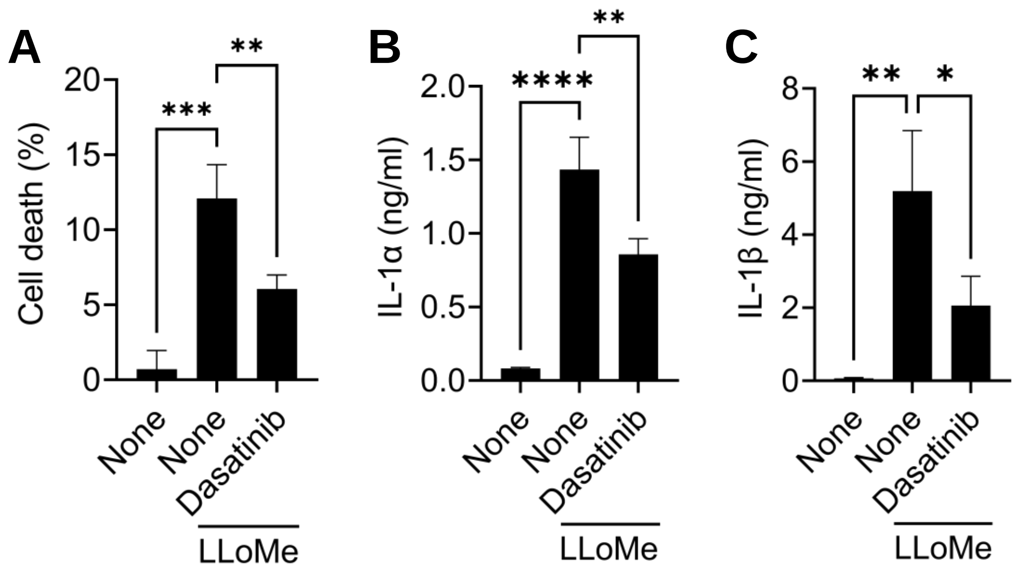

SUPPLEMENTARY FIGURE 3. The effects of dasatinib on pyroptosis induced by L-leucyl-L-leucine methyl ester (LLoMe). **(A-C)** Primed BMDMs were treated with dasatinib (20 μM) and then left unstimulated or stimulated with LLoMe (0.5 mM) for 3 h. **(A)** The cell death rate was determined by measuring lactose dehydrogenase (LDH) activity in the culture supernatants. **(B, C)** Interleukin-1 alpha (IL-1α) and IL-1 beta (β) levels in the culture supernatants were measured using enzyme-linked immunosorbent assay (ELISA). The results are presented as the mean ± SD of values from triplicate wells. *, $P < 0.05$; **, $P < 0.01$; ***, $P < 0.001$; ****, $P < 0.0001$.
